# Rhinovirus-Induced Exacerbations of Asthma and COPD

**DOI:** 10.1155/2013/405876

**Published:** 2013-02-21

**Authors:** Marc B. Hershenson

**Affiliations:** Departments of Pediatrics and Communicable Diseases and Molecular and Integrative Physiology, University of Michigan Medical School, 1150 W. Medical Center Drive, Room 3570B, Medical Science Research Building 2, Ann Arbor, MI 48109-5688, USA

## Abstract

Over the past two decades, increasing evidence has shown that, in patients with chronic airways disease, viral infection is the most common cause of exacerbation. This review will examine the evidence for viral-induced exacerbations of asthma and chronic obstructive lung disease and the potential mechanisms by which viruses cause exacerbations. Attention will be focused on rhinovirus, the most common cause of respiratory exacerbations. Exacerbations due to rhinovirus, which infects relatively few cells in the airway and does not cause the cytotoxicity of other viruses such as influenza or respiratory syncytial virus, are particularly poorly understood. While the innate immune response likely plays a role in rhinovirus-induced exacerbations, its precise role, either adaptive or maladaptive, is debated. Because current treatment strategies are only partially effective, further research examining the cellular and molecular mechanisms underlying viral-induced exacerbations of chronic airways diseases is warranted.

## 1. Introduction

Readers of this paper who care for patients with asthma will be familiar with the following scenario. A patient with recurrent cough and wheeze presents to the clinic for evaluation. The patient states that the only time she has respiratory symptoms is fall, winter, and spring following “colds.” She has no symptoms in the summer, or between colds. She denies she has asthma and does not feel the need to take inhaled steroids even though she has been prescribed them in the past. My response to this patient, and the premise of this review, is that many patients have asthma which is only triggered by viral upper respiratory tract infections. Nevertheless, the role of viral infections in the exacerbation of asthma, chronic obstructive disease (COPD), and other chronic airways diseases was almost completely ignored until the late 1990s, when more sensitive methods of viral detection were developed. While the emphasis of allergic sensitization in the pathogenesis in asthma is strongly justified, serious exacerbations of this disease are more likely to relate to viral infection rather than allergen exposure (Table 1). 

Exacerbations constitute the major cause of morbidity and mortality in asthma and COPD, and therefore vigorous attention towards the problem of exacerbations is warranted. What is the evidence for viral-induced exacerbations of asthma and COPD, and why was the role of viral infection ignored for so long? It was once thought that rhinovirus and other cold viruses did not infect the lower airway. Before the advent of polymerase chain reaction (PCR), viruses were rarely cultured from the bronchoalveolar secretions of immunocompetent individuals, even after experimental infection [1]. Early studies showed that replication of human rhinovirus (RV), the most prevalent respiratory virus, was temperature restricted, with optimal growth below body temperature, at 33°–35°C [2–4]. Exacerbations of chronic airways disease following upper respiratory tract infections were explained by theories linking nasal (upper respiratory tract) and bronchial (lower respiratory tract) disease. The most common explanation was that nasal blockage and mouth breathing lead to inspiration of cold, dry, and unfiltered air, triggering an asthma attack. However, other workers in the field blamed a nasal-bronchial reflex mechanism involving trigeminal and vagal nerves (first proposed in 1919). [5–8]. (This theory was also used to explain how nasal allergies promote asthma.) More recently, release of proinflammatory mediators from the nose and bone marrow into the circulation has been proposed [9]. 

## 2. Evidence That Viral Infections Cause Exacerbations of Asthma and COPD

In contrast to the study showing temperature restriction, subsequent studies showed that many RV strains replicate at body temperature. [4, 10]. For that matter, the temperature of the lower airways may decrease to 33–35°C during periods of increased minute ventilation (i.e., exercise) or cold temperature [11]. However, it was the advent of PCR for the detection of respiratory viruses which really changed the understanding of exacerbations. PCR-based studies examining the prevalence of virus identification among various cohorts of patients with chronic airways disease consistently show a higher prevalence of viral infection during exacerbations. Outpatient children who are sick with asthma exacerbations show anywhere from 62–81% positivity for viral infection versus only 12–41% of children who are well [12, 13]. Picornaviruses (primarily RV) were detected in 65% of cases, coronaviruses in 17%, influenza and parainfluenza viruses in 9%, and RSV in 5% [12]. Similar studies have been performed in hospitalized children, adult outpatients, and hospitalized adults [14–21]. Adults seem to show a slightly lower prevalence of viral infection during exacerbations. Finally, 22 to 64% of patients with COPD exacerbations have virus identified by PCR versus 12 to 19% of nonexacerbating subjects [22–26]. Across all of these studies, RV makes up approximately 50% of the viruses isolated (Table 2). The prevalence of rhinovirus may be even higher depending on the time of year. A recent study detected on RV in 82% of all children admitted to an emergency room for acute asthma between January and July [27]. Together, these studies suggest that viral infections cause exacerbation of asthma and COPD. 

Additional information that viruses indeed cause attacks of chronic airways disease comes from an analysis of emergency department presentations for asthma and COPD over the course of a calendar year. Exacerbations of asthma in children peak after school return from summer vacation (in North America, the first week of September), consistent with an infectious cause [28]. This “epidemic" of asthma exacerbations in children is primarily associated with fall rhinovirus infection. Interestingly, exacerbations of asthma in adults also show elevated risk after school return and around December 25th (the Christmas holiday in Westernized countries), likely due to social interactions with children. Epidemic peaks of COPD exacerbations in adults are observed around the Christmas holiday in Westernized countries, again suggesting an infectious cause such as viral infections transmitted from family members.

**Figure 1:**
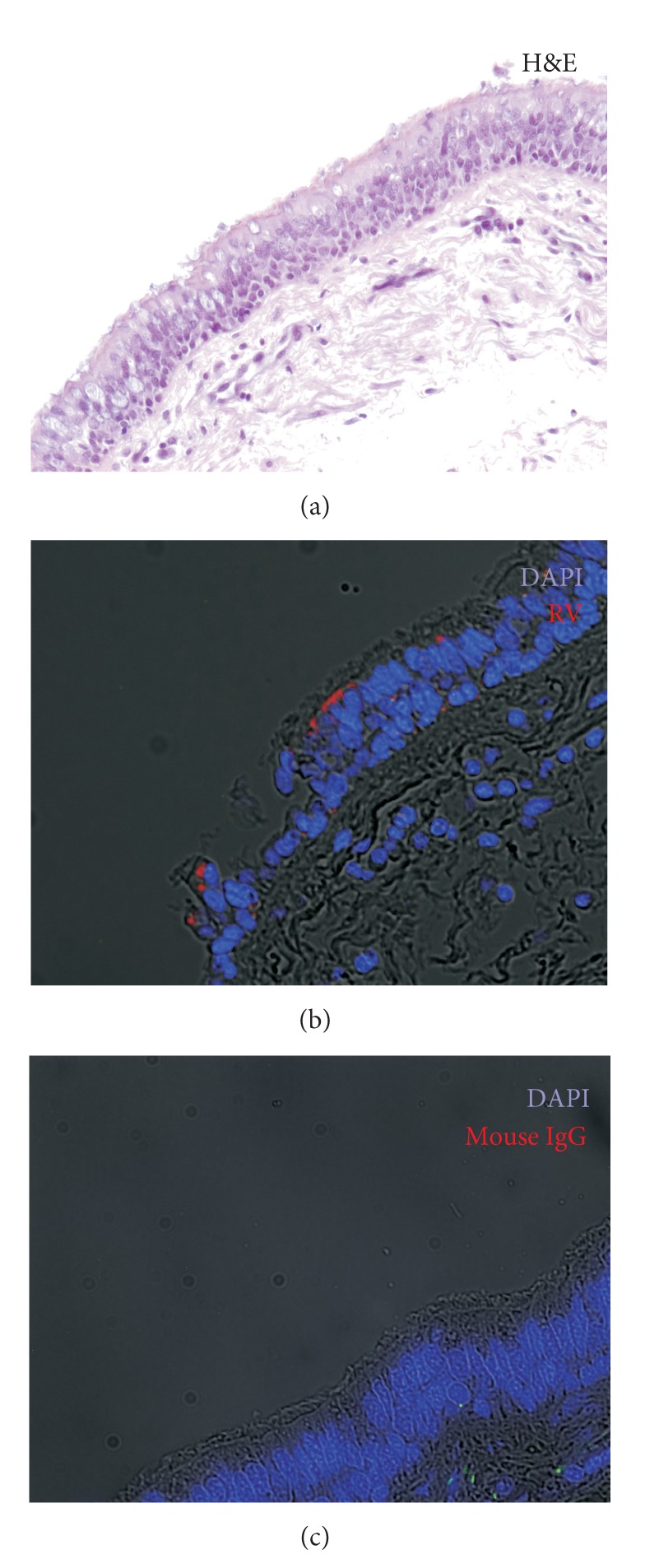
Images of bronchial epithelium from an adult subject without asthma who was experimentally infected with rhinovirus-16. (a) Hematoxylin and eosin stain shows an intact epithelium. (b) Tissue was stained for RV16 capsid protein (red) and DAPI, a nuclear stain. Note that some cells are stained with RV and others are not. (c) Tissue was stained with mouse IgG, a negative control, and DAPI. Note the absence of red staining. Antibody against RV16 and tissue blocks were generously provided by Wai-Ming Lee, Nizar Jarjour, and Jim Gern (University of Wisconsin).

It is now clear that rhinovirus can indeed infect the lower airways. Following experimental infection, RV has been detected in the lower airways by immunostaining, PCR, and *in situ* hybridization for positive-strand viral RNA [29–32]. A study from the University of Wisconsin [30] was highly instructive. These investigators infected adult control and asthmatic subjects with RV16 and then stained biopsy tissue for RV16 capsid protein by immunohistochemistry. RV staining was clearly seen in the cytoplasm of cells in the epithelium (Figure 1). However, staining was patchy and in some samples only 1 or 2 cells were positive. Other investigators have similarly noted that RV infects relatively few cells in the airway [33, 34]. Researchers from Imperial College, London [31] performed *in situ* hybridization of biopsies from experimentally infected patients and found positive-strand viral RNA in the epithelium. Interestingly, there was also sparse staining of cells in the subepithelial layer (there was insufficient detail to determine cell type).

Until recently there was little evidence that rhinovirus replication occurred in the lower airways. However, the Wisconsin group [35], examining sputum of experimentally infected controls and adults, showed persistence of viral RNA up to 14 days after infection; this duration of infection could only occur with viral replication. (Nevertheless, the amount of viral replication in the airways remains uncertain.) Also, patients with asthma who had an exacerbation following experimental infection had higher levels of viral RNA in their sputum compared to asthmatics that did not experience an exacerbation, further evidence that viruses do indeed cause exacerbations.

## 3. Mechanism of Viral-Induced Exacerbations

If indeed viral infections do cause exacerbations of asthma and COPD (and the preponderance of evidence suggests that this is so), what is the proposed mechanism by which viruses cause exacerbations? Rhinovirus, unlike influenza and other viruses, causes minimal cytotoxicity [36, 37]. Also, the amount of epithelial damage does not correlate with the severity of the symptoms, suggesting that symptoms are produced by processes independent of the severity of direct virus-induced damage to the epithelium. While selected studies have demonstrated cytotoxicity in various cell culture models [38], epithelial cell death is considered unlikely to contribute *in vivo* to RV-induced exacerbations of chronic airways disease. 

The current explanation is that rhinovirus infection induces the release of chemokines from airway epithelial cells, thereby attracting inflammatory cells to the airways. In patients with preexisting airway inflammation, the influx of additional inflammatory cells caused by RV infection would lead to additive or synergistic effects and an exacerbation of airways disease. 

There is ample evidence that rhinovirus infection induces airway epithelial cells to express proinflammatory chemokines. Many studies of cultured airway epithelial cells show that RV increases expression of neutrophil-, eosinophil-, and T-cell-attracting chemokines, including CXCL8, CCL5, CCL11, and CXCL10 [10, 39–47]. 

A recent study by Proud and colleagues [48] examined the effect of experimental rhinovirus infection on nasal epithelial cell gene expression *in situ. *The most highly upregulated genes were those encoding chemokines (CCL2, CCL8, CXCL11, CXCL10, CXCL13, CXCL9, and CCL20). We have obtained similar results in the lungs of RV-infected mice (Table 3). Consistent with these results, experimental infections of asthmatic subjects have consistently shown increased number of airway neutrophils, lymphocytes and eosinophils [37, 49–51]. A number of clinical studies suggest that RV potentiates preexisting allergic inflammation [16, 37, 52–55].

However, the notion that viral infection simply augments asthmatic airway inflammation may not explain why asthmatics suffer manifestations of lower airways disease after colds while normal individuals do not. Could asthmatics have a qualitatively different response to rhinovirus infection than controls? Putting another way, does prior allergen sensitization, as occurs in allergic asthma, alter the response to rhinovirus infection? 

Recent studies suggest that the innate immune response to viral infection may be altered in patients with asthma. Why might asthmatics have a different response to RV infection or viral infection in general compared to controls? Airway epithelial damage [56, 57] and mucus metaplasia, both of which occur in asthma, may provide rhinovirus with increased access to more readily infected basal cells [58] and goblet cells [59], thereby increasing viral replication. 

**Figure 2:**
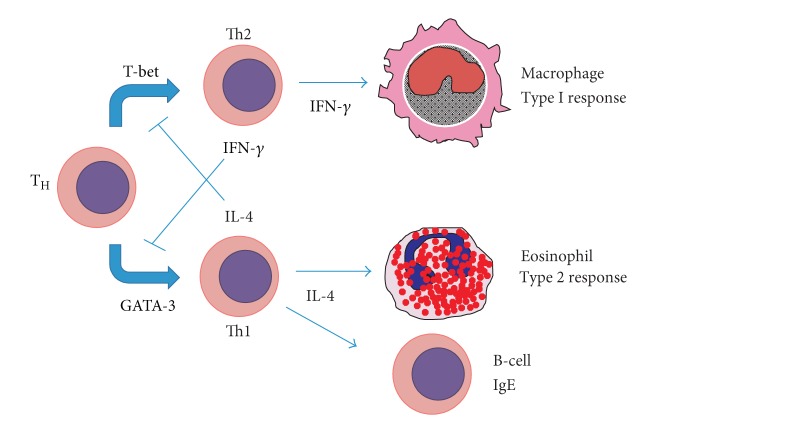
A mechanism to explain defective innate immunity in asthma. The differentiation of Th1 and Th2 T-helper cell lineages is mutually antagonistic. Th2 cells produce IL-4 which blocks Th1 differentiation and Th1 cells produce IFN-*γ* which blocks Th2 differentiation. Thus, individuals with an immune system skewed towards Th2 tend not to produce eosinophils and IgE-producing B cells, but not Th1 cells. The relative lack of IFN-*γ* limits the antiviral response.

Interferon responses, which have been shown to affect the outcome respiratory viral infections in animal models, may also be deficient in patients with asthma. How might this be the case? It is well known that many allergic asthmatics show a skewing of their T cell differentiation towards the Th2 phenotype. Since the differentiation of Th1 and Th2 T-helper cell lineages is mutually antagonistic (e.g., IL-4 blocks Th1 differentiation) [60], allergic asthmatics with Th2 polarization may have a deficiency in their interferon response (Figure 2). During induced colds, asthmatic subjects with strong peripheral blood monocytes and sputum cell interferon gamma responses showed milder cold symptoms and more rapid viral clearance [61, 62]. Also following experimental rhinovirus infection, viral clearance and airway function correlate with blood and bronchoalveolar (BAL) CD4 cell interferon gamma production [63]. Epithelial and BAL cells from asthmatic subjects show reduced type I and type III interferon responses to rhinovirus infection *ex vivo* [64, 65]. Together, these data suggest that asthmatics may be more susceptible for RV infection. 

On the other hand, other investigators have not found differences in interferon expression or viral clearance between controls and asthmatics. Two groups have failed to show reduced interferon responses in cultured airway epithelial cells from subjects with asthma [66, 67]. Perhaps most importantly, a difference in viral copy number or titer between controls and asthmatics has not been demonstrated following experimental RV infection [35].

To address the question of whether preexisting allergic airways disease alters the response to viral infection, and whether the interferon response to rhinovirus is required for viral clearance (or part of a maladaptive response to a relatively innocuous infection), we developed a mouse model of human RV infection [68–73]. Before describing this model, however, I like to say a little bit about the basic biology of rhinovirus. RV is an RNA virus from the *Picornaviridae* family. Rhinovirus is composed of a single strand of positive-sense RNA enclosed in a small icosahedral capsid. There are greater than 100 known serotypes of rhinovirus. The major subgroup (88 serotypes) utilizes ICAM-1 as a receptor [74]; the minor group (11 serotypes) uses the family of low density lipoprotein receptors (LDL-R, VLDL-R, and LDL-R related protein) [75]. Other surface molecules, including TLR2, may serve as a coreceptor and also be required for viral responses [76]. Rhinoviruses may also be classified not only on the basis of their host receptor but according to their resistance to antiviral drugs. According to this classification, there are 74 type A viruses and 25 type B viruses [77]. A third group of rhinoviruses, type C, has recently been discovered [78–80]. Rhinoviruses have also been classified according to their genome sequences [81]. Interestingly, RV16, a major group virus commonly used for experimental human infection, and RV1, which has been used in animal models of rhinovirus infection (see below), are closely related. At this time, the only cell type conclusively shown to be infected by RV in humans is the airway epithelial cell. Typically, RV infects small clusters of cells in the epithelial layer [30]. Rhinovirus is internalized by clathrin-mediated endocytosis [82–85]. The endosome acidic pH triggers viral uncoating and RNA insertion. RV replication occurs entirely in the cytoplasm. During viral replication, negative sense RNA and double-stranded RNA intermediates are formed. dsRNA produced during viral infection represents an important stimulus for the host innate immune response. dsRNA is recognized and engaged by three pattern recognition receptors, toll-like receptor (TLR)-3, localized to the endosomal and plasma membranes, and cytoplasmic proteins (RIG)-I, and MDA-5 which are intracellular receptors for viral dsRNA [86, 87]. 

 Returning to the problem of animal models, major group viruses do not bind to murine cells, owing to a lack of homology between the human and mouse ICAM-1. However, it was subsequently shown that minor group viruses infect LA-4 mouse tracheal epithelial cells [88]. We [68–72, 89] and others [90] therefore infected mice with a minor group virus, RV1B, by the intranasal route. Because of as-yet undefined factors which limit viral replication in the mouse, infection is followed by a steady reduction in viral copy number and titer. However, careful studies of viral copy number show a spike in positive-strand vRNA approximately 24 hours after infection and a small amount of negative-strand vRNA indicating viral replication. We also found a robust interferon response to infection, implying viral replication. Immunofluorescence showed infection of airway epithelial cells and, interestingly, occasional subepithelial cells resembling monocytes. Airways of infected mice showed neutrophilic inflammation which decreased with time, as well as subsequent lymphocytic infiltration, a pattern classically associated with viral infection. When we examined respiratory system resistance changes in response to methacholine administration, we found a small but significant increase in airways responsiveness in rhinovirus-infected mice which persisted at least four days after infection. Interestingly, mice infected with UV-irradiated replication-deficient virus showed modest airway inflammation and responsiveness one, but not four, days after infection. These data suggest that rhinovirus could induce a state of airways hyperresponsiveness without viral replication in the lungs, at least under some circumstances. Consistent with this, UV irradiation inhibits but does not abolish major group RV-induced IL-8 release in cultured airway epithelial cells [43, 91, 92]. 

**Figure 3:**
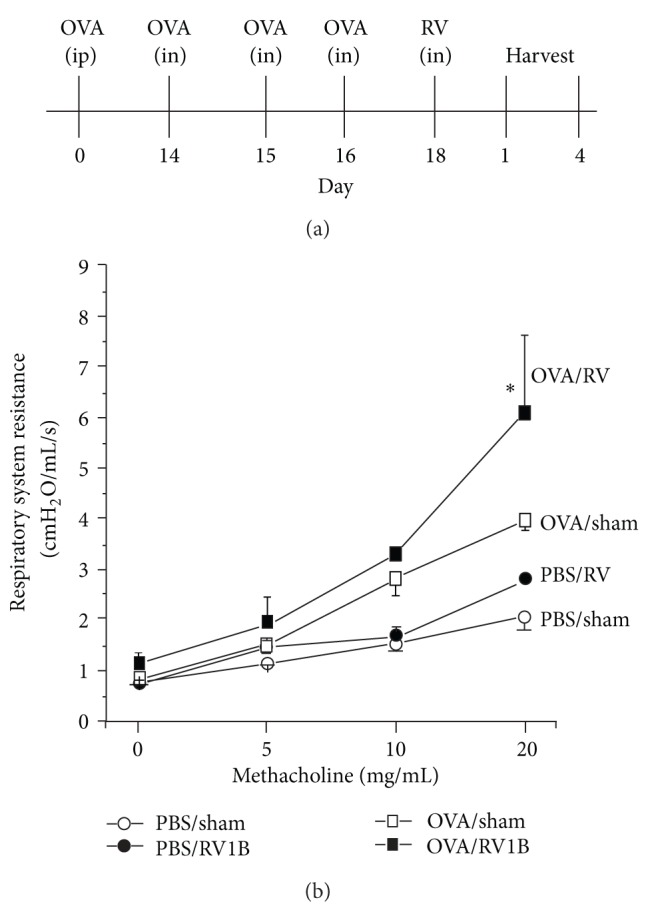
A mouse model of RV-induced asthma exacerbation. (a) Mature mice were sensitized with ovalbumin (OVA) and alum given intraperitoneally. This was followed by intranasal challenge with additional ovalbumin. One day after the last OVA challenge, animals were infected with RV1B, a minor group virus capable of infecting mouse cells. (b) Ovalbumin and RV1B have synergistic effects of airways responsiveness. Airways responsiveness was assessed by measuring changes in respiratory system resistance in response to increasing doses of nebulized methacholine. Respiratory system resistance was measured in anesthetized and endotracheally intubated mice using a computerized ventilator (Scireq, Montreal, Quebec, Canada).

 We next set out to develop a model of viral-induced asthma exacerbation [72]. We employed a commonly used model of allergic airways disease. Mice were treated with intraperitoneal and intranasal ovalbumin and then infected with rhinovirus. Mice treated with ovalbumin alone demonstrated a significant increase in airways responsiveness, with respiratory system resistances higher than those obtained after rhinovirus infection. When we combined ovalbumin and rhinovirus treatments, airways hyperresponsiveness increased significantly over that generated by either ovalbumin or rhinovirus alone (Figure 3). This state of airways hyperresponsiveness persisted at least 4 days after infection. When we examined airway inflammation, we found additive or synergistic increases in airway neutrophils, eosinophils, macrophages, lymphocytes, and chemokines such as CCL11 and CCL2. We also found that the combination of ovalbumin and rhinovirus caused additive increases in the Th2 cytokines IL-4 and IL-13. 

 When we performed immunohistochemistry to look for the source of CCL11 production, we expected to find CCL11 expression in rhinovirus-infected airway epithelial cells. However, to our surprise, we found that the main source of CCL11 was airway mononuclear cells. When we performed fluorescence microscopy using labeled antibodies against rhinovirus, CCL11 and CD68, a macrophage surface marker, we confirmed ample colocalization of RV1B, CCL11, and CD68, indicating CCL11 expression by CD68-positive macrophages. While most cells were in the subepithelium, we also found CD68-positive macrophages in the epithelial layer and the airway lumen. In follow-up studies, we also found colocalization of CD68, RV1B, and IL-4 in subepithelial cells. Several studies have examined the infection of monocytic cells by rhinovirus *in vitro *[93–98]. However, the former data represent the first demonstration of rhinovirus infection of monocytes *in vivo*. At this point, we do not know whether colocalization represents replicative infection of airway macrophages, or simple phagocytosis of the virus. A small amount of viral replication has been noted in RV-infected peripheral blood monocyte-derived macrophages, but not in BAL-derived macrophages [93, 94]. 

 To address this further, we isolated bronchoalveolar lavage macrophages from control and ovalbumin-sensitized and -challenged mice and infected them with rhinovirus *ex vivo*. Macrophages from control mice showed ample Th1 cytokine (TNF-*α*, IL-12p70) expression in response to RV infection, but little or no Th2 cytokine (IL-4, IL-13, CCL11) production. In contrast, BAL cells from ovalbumin-sensitized and -challenged mice showed brisk Th2 responses to *ex vivo* infection, but decreased Th1 responses. This pattern of cytokine expression was consistent with a change in phenotype from M1 to alternatively activated M2 macrophages [99]. We confirmed this by measurement of M2 markers such as arginase-1, YM-1, and MGL-2. Furthermore, flow cytometry showed high levels of CD11b expression, indicative of exudative macrophages found after lung infection or injury. To determine the requirement of these macrophages for the observed airways hyperresponsiveness of rhinovirus-infected and ovalbumin-sensitized and -challenged mice, we depleted the macrophages using clodronate liposomes. Administration of clodronate decreased the number of airway macrophages, but not neutrophils or lymphocytes. However the lungs of mice receiving clodronate showed reduced levels of CCL11, eosinophils, IL-13, and airways responsiveness, demonstrating that macrophages are responsible for RV-induced airway responses in mice with preexisting allergic airways disease. The production of Th2 cytokines by lung macrophages is consistent with recent work showing that, in addition to Th2 helper T cells, innate immune cells may contribute to the production of Th2 cytokines in asthma [100–107]. Finally, our unpublished experiments indicate that, under certain circumstances, M1-polarized macrophages may also be targeted by RV infection. Taken together, our data suggest that, rather than causing epithelial infection or damage, RV elicits asthma exacerbations by infecting inflammatory cells, in particular cells of the monocyte/macrophage lineage. 

 But is there any evidence that rhinovirus infects inflammatory cells in humans? With the help of Jim Gern, Nizar Jarjour, and others from the University of Wisconsin, we have begun to address this question. We obtained biopsy specimens from control and asthmatic subjects experimentally infected with rhinovirus. Early unpublished results indicate colocalization of RV and CD68 in epithelial and subepithelial cells. 

 Returning to the question of antiviral innate immunity in asthma, we also employed our animal model to address this issue. Does the inflammatory response to rhinovirus promote viral clearance and prevent more severe viral-induced disease? Or is the inflammatory response to rhinovirus a maladaptive response to an innocuous stimulus, leading to exacerbations of lower airways disease? These questions have important ramifications for treatment. One often hears the statement that individuals “need to boost their immune response” to fight off colds or cold-induced asthma attacks. The Imperial College group has championed the idea that the interferon response is deficient in asthmatics [64, 65], thereby explaining viral-induced exacerbations. In this case, boosting of the immune response with interferons might be helpful. 

 To test the hypothesis that the immune response to rhinovirus may be counterproductive, we infected mice with defects in TLR and MDA-5 [89], molecules which recognize double-stranded RNA on the cell surface or in the cell cytoplasm, respectively. As noted above, these signaling pathways are responsible for double-stranded RNA-stimulated interferon production. Our previous work showed that MDA-5, an RNA helicase, is required for rhinovirus-induced interferon production in cultured airway epithelial cells [73]. TLR3 knockout mice had normal interferon responses to rhinovirus infection. However, MDA5 knockout mice showed a delay in type I interferon responses and a significant and persistent reduction in type III interferon lambda production. However, despite these deficiencies, MDA5 knockout mice showed only small increases in RV vRNA and titer. Further, RV-infected knockout mice showed less, rather than more, airway inflammation. Similar results were found in rhinovirus-infected ovalbumin-sensitized and -challenged knockout mice. These data call into question the interferon requirement for RV clearance and suggest that the proinflammatory response to rhinovirus infection is indeed maladaptive. Indeed, a recent study found that interferon-lambda concentrations were higher in rhinovirus-infected infants with wheezing compared to those without wheezing [108].

## 4. Viral-Induced COPD Exacerbations

 What about viral-induced COPD exacerbations? Since COPD differs from asthma in several key respects, the pathogenesis of virus induced exacerbations may be different in this disease. COPD differs from asthma in at least the following ways. The key trigger for COPD is cigarette smoke rather than aeroallergens, air pollutants, or infection. COPD encompasses two distinct processes, chronic bronchitis, and emphysema, which result in structural changes that limit airflow. The COPD airway epithelium undergoes squamous metaplasia. In COPD, macrophages and CD8-positive cell play key roles rather than Th2 cells, eosinophils, or mast cells. COPD pathogenesis appears to be dominated by TGF-*β*, TNF-*α*, IL-1*β*, and IL-6. The lower airways of 25 to 50% of patients with COPD are colonized by bacteria, particularly *Haemophilus influenzae, Streptococcus pneumonia*,and *Moraxella catarrhalis. *[109, 110]. Finally, oxidative stress resulting either from inhaled oxidants or inflammatory cells plays a significant role. As mentioned above, about one half of COPD exacerbations are associated with viral infections, the majority of which are due to RV [22–26]. COPD exacerbations may also be associated with the isolation of the new bacterial strain [110]. Thus, interactions between virus, bacteria, and host may play a role in the pathogenesis of COPD exacerbations.

 Recent studies by the Imperial College London, in which patients with COPD were experimentally infected with rhinovirus [111], have provided new information about RV-induced exacerbations in these patients. Compared to controls, COPD patients show increased sputum neutrophils in response to experimental RV infection. Other investigators have shown increased neutrophils, eosinophils, macrophages, and lymphocytes in the airways of COPD patients experiencing exacerbations [112–118]. Unlike asthma patients, COPD patients experimentally infected with RV show increased sputum viral copy number versus controls, and viral load correlates with sputum neutrophils and interleukin-8 levels [111]. BAL cells from patients with COPD show reduced interferon-alpha and -beta responses to rhinovirus infection *ex vivo*. Together, these results provide ample evidence of altered innate immunity against viruses. These data reinforce *in vitro* data showing that exposure of lung cells to cigarette smoke extract reduces CXCL10, interferon-beta, and RIG-I expression in response to influenza virus infection [119].

 Viral infection may also attenuate the antibacterial innate immune response in COPD. For example, rhinovirus exposure impairs immune responses to bacterial products in human alveolar macrophages. We have shown that RV infection of mucociliary-differentiated airway epithelial cells cultured at air-liquid interface causes barrier dysfunction which allows translocation of *Haemophilus influenzae *across the epithelium [69]. We have also shown that elastase- and LPS-treated mice with a lung phenotype similar to COPD show delayed clearance of rhinovirus *in vivo,* similar to humans with COPD [71].

Cystic fibrosis is a chronic airways disease with similarities to asthma and COPD. CF may also be exacerbated by viral infection. For example, viruses were detected in 46% of patients with exacerbations of cystic fibrosis, compared to only 18% of patients in stable condition [120]. RV has been identified in 13 to 58% of cystic fibrosis patients with acute respiratory illness and was associated with increased respiratory symptoms, worse airway function and secondary bacterial infection, compared to uninfected patients [120–123].

## 5. Rhinovirus-Associated Wheezing in Early Infancy: Exacerbations of Preexisting Airways Disease or a Factor in Asthma Development? 

Over the past decade, data from a birth cohort of high-risk infants with a positive family history of asthma (nicknamed the Childhood Origins of ASThma, or COAST) have shown that wheezing-associated illness with RV is the most important risk factor for asthma development [124, 125]. The relative risk of asthma development in infants with wheezing associated with rhinovirus infection was far higher than that of infants with allergen sensitization or RSV infection alone. Also, in infants hospitalized for respiratory infection-associated wheezing, infection with RV was associated with asthma development [126] in contrast to respiratory syncytial virus (RSV), which was negatively associated [127]. Together, these data suggest two possibilities, either that RV infection causes asthma, or that RV infections may simply reveal a preexisting tendency for asthma. More recent data suggest that the latter is likely; prospective characterization of the COAST birth cohort demonstrated that allergic sensitization precedes HRV wheezing and that the converse is not true [128]. Also, it was recently shown that children developing asthma by age seven had a lung function deficit and increased bronchial responsiveness as neonates [129], suggesting that asthma precedes RV infection. If this is the case, then wheezing-associated illnesses due to rhinovirus are essentially viral-induced asthma exacerbations. 

Is it possible that, under the right circumstances, for example, the appropriate genetic background and allergen exposure, rhinovirus infection in early life modulates the immune response, increasing the likelihood of asthma development? A positive family is a known risk factor for asthma development, and it has recently been found that infants of mothers with asthma are more likely to have severe respiratory tract infections with RV [130]. To address this possibility, we infected baby mice with rhinovirus at 2 to 3 days of life. Unlike mature mice, rhinovirus-infected neonatal mice showed mucus metaplasia and airways hyperresponsiveness which lasted for one month after infection [131]. This is reminiscent of data from Washington University St. Louis in mature mice following infection with Sendai virus [132]. However, the finding of a developmental difference in response to rhinovirus, an infection that does not cause cytotoxic effects, warrants further investigation. Studies using neutralizing antibody and an IL-4 receptor knockout mouse showed that the effect of RV was dependent on IL-13. We also found increased production of IL-13 by invariant natural killer T (iNKT) cells. 

The notion that early rhinovirus infection could modulate future immune responses, leading to allergic airway inflammation, has been bolstered by the discovery of novel epithelial-derived cytokines which promote Th2 differentiation. These cytokines, thymic stromal lymphopoietin (TSLP), IL-25, and IL-33, play a key role in the maturation of Th2 cells via dendritic cell (DC) activation [133–138], as well as the activation of mast cells and iNKT cells [139]. Moreover, TSLP, IL-25, and IL-33 induce IL-13 production by two novel innate effector leukocytes that mediate Th2 immunity—type 2 innate lymphoid cells (also called natural helper cells or nuocytes)[100–106] and type 2 myeloid cells [107]. 

## 6. Conclusions

 Respiratory viral infections cause exacerbations of asthma and COPD. This is evidenced by the increased identification of viruses in the respiratory tract during exacerbations compared to stable periods. Also, epidemic cycles of asthma and COPD exacerbations occur after school return and around the Christmas holiday, consistent with an infectious cause. It has also been well demonstrated that viruses infect and replicate in the lower airways. Finally, the severity of viral-induced exacerbations correlates with viral load. Viral infection of the epithelium increases expression of chemokines, leading to influx of inflammatory cells and increased airway inflammation. Innate immunity to viral infection may be decreased in asthma, owing to epithelial damage, mucus metaplasia, and immune system polarization towards a Th2 phenotype. Disease exacerbation in asthmatics (rather than a simple cold) may be explained in part by infection of airway inflammatory cells, which are increased in patients with preexisting airways disease. As with asthma, viral-induced COPD exacerbations are associated with increased airway neutrophils, eosinophils, and lymphocytes. Patients with COPD demonstrate clear evidence of impaired antiviral innate immune responses. Finally, in COPD, viral infection may suppress antibacterial responses and increase translocation of bacteria across the airway epithelium, leading to exacerbation and perhaps disease progression.

## Figures and Tables

**Table 1:** Causes of asthma exacerbation.

(1) Viral infection (most commonly rhinovirus)	
(2) Allergen exposure (tree, grass, and weed pollens; mold; animal dander; dust mites; and cockroach particles)	
(3) Exercise	
(4) Irritants including environmental tobacco smoke exposure, smoke from wood-burning appliances or fireplaces, strong odors from perfumes, cleaning agents, air pollution, and occupational dust or vapors	
(5) Medications (aspirin and other anti-inflammatory drugs, and beta blockers)	
(6) Changes in weather (cold air, changes in temperature, and humidity)	
(7) Gastroesophageal reflux	
(8) Sinusitis	

**Table 2:** Respiratory viruses.

Human rhinovirus
Coronavirus
RSV
Influenza virus
Adenovirus
Parainfluenza
Human metapneumovirus
Bocavirus
Enterovirus

**Table 3:** Fold regulation of mouse lung genes following infection with human rhinovirus. Ten most upregulated genes are shown. Eighty-six total genes encoding chemokines, chemokine receptors, interleukins, interleukin receptors, other cytokines, and other cytokine receptors were monitored by PCR array (SABiosciences, Valencia, CA, USA).

Gene	Fold regulation
Cxcl10	41.4574
Ccl7	20.7436
Cxcl5	18.1904
Ccl2	11.5655
Cxcl11	9.2016
Ccl12	8.8608
Ccl8	6.0771
Tlr2	5.6126
Ccl4	5.2671
Cxcl2	4.3805
